# Effects of early feeding on the host rumen transcriptome and bacterial diversity in lambs

**DOI:** 10.1038/srep32479

**Published:** 2016-08-31

**Authors:** Weimin Wang, Chong Li, Fadi Li, Xiaojuan Wang, Xiaoxue Zhang, Ting Liu, Fang Nian, Xiangpeng Yue, Fei Li, Xiangyu Pan, Yongfu La, Futao Mo, Fangbin Wang, Baosheng Li

**Affiliations:** 1 College of Animal Science and Technology, Gansu Agricultural University, Lanzhou 730000, P. R. China; 2 College of Pastoral Agriculture Science and Technology, Lanzhou University, Lanzhou 730000, P. R. China; 3 Engineering Laboratory of Sheep Breeding and Reproduction Biotechnology in Gansu Province, Minqin 733300, P. R. China; 4 JinchangZhongtian Sheep Industry Co. Ltd., Jinchang 737100, P. R. China

## Abstract

Early consumption of starter feed promotes rumen development in lambs. We examined rumen development in lambs fed starter feed for 5 weeks using histological and biochemical analyses and by performing high-throughput sequencing in rumen tissues. Additionally, rumen contents of starter feed-fed lambs were compared to those of breast milk-fed controls. Our physiological and biochemical findings revealed that early starter consumption facilitated rumen development, changed the pattern of ruminal fermentation, and increased the amylase and carboxymethylcellulase activities of rumen micro-organisms. RNA-seq analysis revealed 225 differentially expressed genes between the rumens of breast milk- and starter feed-fed lambs. These DEGs were involved in many metabolic pathways, particularly lipid and carbohydrate metabolism, and included *HMGCL* and *HMGCS2*. Sequencing analysis of 16S rRNA genes revealed that ruminal bacterial communities were more diverse in breast milk-than in starter feed-fed lambs, and each group had a distinct microbiota. We conclude that early starter feeding is beneficial to rumen development and physiological function in lambs. The underlying mechanism may involve the stimulation of ruminal ketogenesis and butanoate metabolism via *HMGCL* and *HMGCS2* combined with changes in the fermentation type induced by ruminal microbiota. Overall, this study provides insights into the molecular mechanisms of rumen development in sheep.

The rumen has several important physiological functions, including absorption, transport, metabolic activity, and host protection^1^. The rumen of a new-born ruminant is essentially non-functional, as it has a smooth, stratified, squamous epithelium with no prominent papillae, and the microbiota has not yet been established^2^,^3^. The initiation of solid feed intake can trigger development of the rumen. The fermentation of solid feed by ruminal microorganisms results in the production of volatile fatty acids (VFAs)^4^,^5^. Intraruminal VFA administration stimulates the morphological development of the rumen epithelium in milk-fed animals^3^,^6^,^7^. Feeding starter along with milk is a common method for accelerating early rumen development^8^. It has been reported that solid feed consumption can increase total VFA concentrations and the molar proportion of acetate and butyrate in the rumen of starter + milk replacer-fed lambs when compared with lambs fed only milk replacement during 1 to 84 days of life^9^. Indeed, administering grain-based feed and orchard grass hay to neonatal ruminants significantly increase the length and density of the rumen papillae, whereas feeding milk alone resulted in little development of these structures and poor animal growth^10^. Therefore, this feeding strategy provides biologically appropriate fuels, and is thought to enhance early rumen development. However, the molecular mechanisms involved in this process remain unclear.

Daichi *et al*. identified three genes (*HMGCS2*, *AKR1C1* and *FABP3*) associated with rumen development by screening for candidate genes by *in silico* digital differential display (DDD)^11^. *TGFβ1* (transforming growth factor-beta 1) and *ESRRα* (oestrogen-related receptor alpha) can contribute to the development of the rumen epithelium and energy metabolism, respectively, as transcriptional regulators. These factors also trigger activation of downstream targets that can mediate rumen development and function in growing calves^12^. Changes of monocarboxylate transporter 1 (*MCT1*) expression occur in an age-dependent manner in the rumen epithelium of neonatal calves, which suggests that the expression of *MCT1* may be affected by the provision of liquid feed^13^. During the studies described above, many changes in gene expression related to rumen development were identified in calves.

Recently, interest in the diversity and function of ruminal bacteria has grown. The ruminal bacteria digest both complex and simple carbohydrates in the rumen and produce nutrients, such as volatile fatty acids (VFA), microbial proteins, and vitamins, for the host. Diet is a major factor that influences the structure and function of microbial communities that are present in the rumen^14^. The effect of diet on the structure of rumen microbial communities has been widely investigated using both culture-based and standard molecular methods, such as PCR, PCR-DGGE, and real-time PCR^15^,^16^,^17^,^18^,^19^,^20^,^21^,^22^,^23^,^24^,^25^. Currently, next-generation sequencing is widely used to study rumen microbial ecology. The application of such an approach would allow for much greater coverage of the microbial diversity and physiology of a complex environment, such as the rumen in calves^26^, cows^27^,^28^, and goats^29^.

In this present study, next-generation sequencing (RNA-seq and 16S rRNA gene sequencing) was used to assess the effects of early feeding on host rumen gene expression and microbial community structure in lambs. We identify 225 differentially expressed genes between starter feed- and breast milk-fed lambs. These differentially expressed genes (DEGs) are involved in many metabolic pathways, such as carbohydrate and lipid metabolism. We also elucidate diet-dependent rumen microbial taxonomical composition changes at the phylum and genus levels. Our findings offer insights into the complexity of rumen development in sheep.

## Results

### Growth performance, rumen morphology, rumen fermentation parameters, and enzymatic activity

Growth performance, rumen morphology, rumen fermentation parameters, and the enzymatic activity of rumen microorganisms from the beast milk- and starter feed-fed groups are shown in Table 1. The body weight (BW) and average daily gain (ADG) were higher in the starter feed- than in the breast milk-fed group. For rumen morphology, starter feed did not affect the papilla width (PW) or muscular thickness (MT) of the rumen. The weight of the reticulo-rumen (WRR), the volume of the reticulo-rumen (VRR), and the papilla height (PH) of the rumen were higher in the starter feed-fed group than in the breast milk-fed group. Regarding the rumen fermentation parameters, the starter feed-fed group had higher concentrations of total VFA, propionate, butyrate, and valerate, and lower concentrations of acetate, isobutyrate, and A:P compared with the breast milk-fed group. Concentrations of nitrogen, ammonium nitrogen, urea nitrogen, and protein nitrogen were higher in the starter feed-fed group than in the breast milk-fed group. For the enzymatic activity potentials of rumen contents, proteinase activity in the starter feed-fed group was lower than in the breast milk-fed group. The amylase and carboxymethylcellulase activities in the starter feed-fed group were higher than in breast milk-fed group.

### RNA sequencing—data mapping and annotation

A total of 6 cDNA libraries were sequenced from the rumen of starter feed- and breast milk-fed groups (n = 3 per group). Libraries were sequenced using a HiSeq 2500 sequencing platform at Personalbio-Shanghai, China, and 6 sets of reads were obtained. After removing adaptors and filtering, we obtained a total of 149.9 M and 123.3 M high-quality clean reads for the breast milk- and starter feed-fed groups, respectively. RNA-seq yielded 24.6 M to 68.7 M reads for all 6 samples, and more than 90% of reads met our quality control criteria (Supplementary Table S1).

After mapping clean reads to the ovine genome, 68.81–84.24% of reads were successfully aligned, and 94.38–95.68% of mapped reads had unique genomic locations. Moreover, 64.95–78.05% of reads mapped to a gene, 87.29–93.10% of reads mapped to an exon, and 21.95–35.05% of reads mapped to introns or intergenic regions (Supplementary Table S2 and Figure S1).

### Differentially expressed genes between starter feed- and breast milk-fed lambs

**Figure 1:**
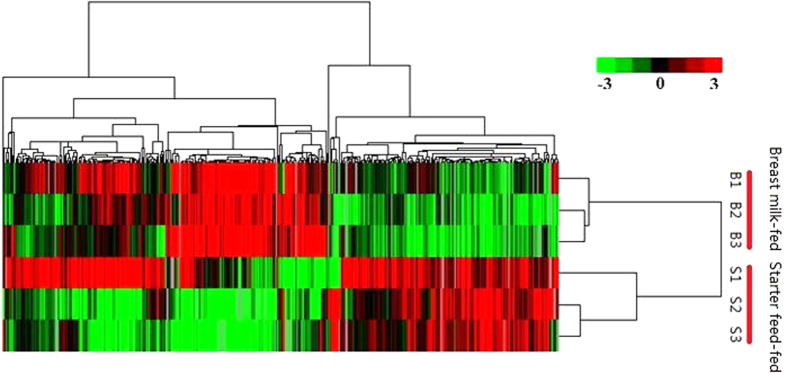
Average linkage hierarchical clustering analysis of the log_2_ transformed change ratio of 18,716 unigenes generated with Cluster 3.0 software. Data are shown in a tree analysis generated using the software package Java Treeview. Each row represents a differentially expressed gene and each column represents a sample. Green and red colour gradients indicate a reduction or increase in transcript abundance, respectively.

In this present RNA-seq study, 18,716 genes were detected in the rumen of all 6 individuals (Supplementary Table S3). To characterize the relationship of genome-wide expression profiles between starter feed- and breast milk-fed lambs, all annotated unigenes from 6 rumen samples were clustered using the software Cluster 3.0 (Fig. 1). The genes detected in the different feeding treatment groups were clearly distinct. A total of 225 genes were differentially expressed, with a criteria of at least a 2-fold difference and a P-value less than 0.05 (|log2FC| ≥ 1, p < 0.05, FDR < 0.05), among which 188 and 37 genes were up- and down-regulated in the starter feed-fed group (Supplementary Table S4). Table 2 shows the top 20 differentially expressed (DE) genes, including the top 10 genes with either higher or lower expression in the starter feed-fed compared to the breast milk-fed lambs.

**Figure 2:**
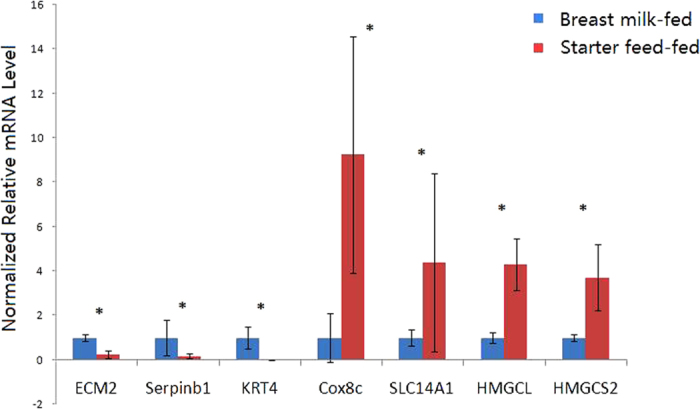
Validation of differentially expressed genes in the rumen from breast milk-and starter feed-fed lambs. The qPCR measurements of the expression of *Cox8c*, *SLC14A1*, *Serpinb1*, *ECM2*, *KRT4*, *HMGCL*, and *HMGCS2* mRNA transcripts were analysed using the ΔΔCt method; *significant difference between the groups of lambs.

To validate the differential expression of genes, we selected seven genes for qRT-PCR analysis. Compared with the breast milk-fed group, expression of *ECM2*, *Serpinb1*, and *KRT4* mRNA transcripts were lower in rumens of starter feed-fed lambs, whereas the expression of *Cox8c*, *SLC14A1*, *HMGCL*, and *HMGCS2* mRNA transcripts were higher in the rumens of starter feed-fed lambs (Fig. 2). Expression of 7 selected genes (*ECM2*, *Serpinb1*, *KRT4*, *Cox8c*, *SLC14A1*, *HMGCL*, and *HMGCS2*) showed significant differences between the breast milk- and starter feed-fed groups. Accordingly, the qRT-PCR analyses largely confirmed the RNA-seq findings, with the correlation coefficient of the fold-change (FC) values from the two methods being 0.99 and the R^2^ value for the linear regression also being 0.99, indicating the robustness of the RNA-seq data.

### Pathway analysis of differentially expressed genes

**Figure 3:**
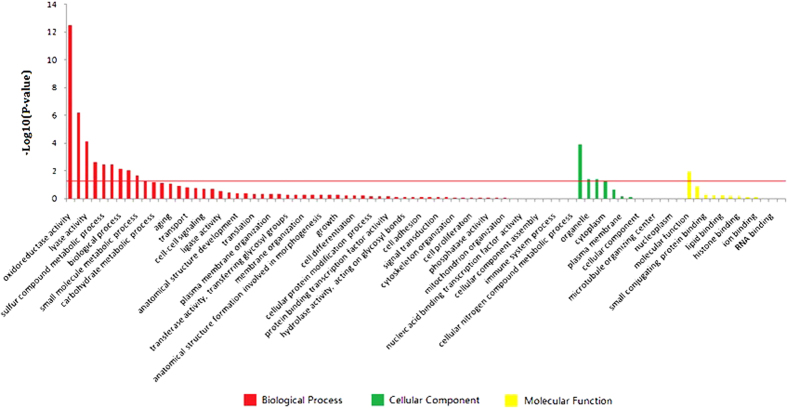
Gene ontology (GO) classification of differentially expressed genes. The red line indicates *P* = 0.05. Details of the GO enrichment analysis are presented in Supplementary Table S5.

To explore the biological functions of the 225 DEGs, GO (Gene Ontology) enrichment analysis was performed. These DEGs were classified into three main categories: biological process, cellular component, and molecular function (Fig. 3 and Supplementary Table S5). Among all 86 GO terms, “Oxidoreductase activity” (*P* = 3.12E-13) was the most enriched cluster, followed by “Lipid metabolic process” (*P* = 6.05E-07), “Lyase activity” (*P* = 8.00E-05), “Cofactor metabolic process” (*P* = 2.28E-03), “Sulfur compound metabolic process” (*P* = 3.37E-03), “Transferase activity, transferring acyl groups” (*P* = 3.40E-03), “Biological process” (*P* = 7.53E-03), “Isomerase activity” (*P* = 9.37E-03), “Small molecule metabolic process” (*P* = 2.29E-02), “extracellular region” (*P* = 1.21E-04), “organelle” (*P* = 4.16E-02), “extracellular space” (*P* = 4.29E-02), and “molecular function” (*P* = 1.15E-02).

**Figure 4:**
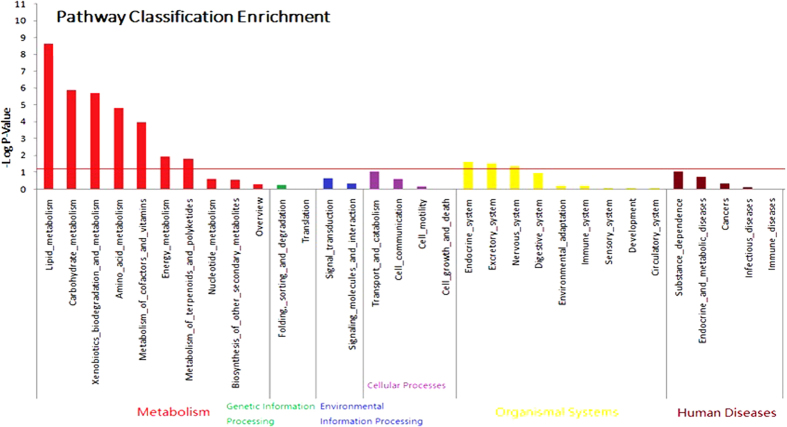
KEGG enrichment analysis of differentially expressed genes. The red line indicates *P* = 0.05. Details of the KEGG enrichment analysis can be found in Supplementary Table S6.

KEGG (Kyoto Encyclopaedia of Genes and Genomes) enrichment analysis was used to align all DE genes to two specific pathways—Metabolism and Organismal Systems (Fig. 4 and Supplementary Table S6). The most enriched category was lipid metabolism (*P* = 2.42E-09, 331 genes, including 19 DEGs), followed by carbohydrate metabolism (*P* = 1.27E-06, 337 genes, including 16 DEGs), xenobiotic biodegradation and metabolism (*P* = 2.08E-06, 102 genes, including 9 DEGs), amino acid metabolism (*P* = 1.47E-05, 237 genes, including 12 DEGs), metabolism of cofactors and vitamins (*P* = 1.05E-04, 130 genes, including 8 DEGs), energy metabolism (*P* = 1.14E-02, 121 genes, including 5 DEGs), metabolism of terpenoids and polyketides (*P* = 1.68E-02, 18 genes, including 2 DEGs), the endocrine system (*P* = 2.32E-02, 760 genes, including 15 DEGs), the excretory system (*P* = 2.89E-02, 104 genes, including 4 DEGs), and the nervous system (*P* = 4.68E-02, 555 genes, including 11 DEGs).

### Rumen microbial 16S rRNA sequencing data and alpha diversity

After data filtering, quality control, assembling of pair-end reads, and the removal of primers, chimeras, and low confidence singletons, a total of 189,357 V1-V3 16S rRNA sequence reads from 12 samples, with an average of 15,780 sequence reads for each sample (the minimum and maximum values for one sample were 11,153 and 25,008 sequence reads, respectively), were used in this study. The average length of sequence reads after primer removal was 395 bp. The overall number of OUTs detected by our analysis was reached 6798 based on 97% nucleotide sequence identity between reads.

The rarefaction curve was constructed by random sampling for all sequences, which (Supplementary Figure S2) revealed that most of our sampling efforts yielded sufficient OUT coverage to accurately describe the bacterial composition of each group of lambs. Alpha-diversity measures (Table 3) indicated that the Chao, ACE, and Shannon index of the starter feed-fed lambs were higher than those of the breast milk-fed lambs (*P* < 0.01), while the Simpson index of the starter feed-fed lambs was lower than that of breast milk-fed lambs (*P* < 0.05).

### Taxonomic composition of ruminal bacterial communities between starter feed- and breast milk-fed lambs

In total, 21 phyla were identified within the ruminal microbiota. The abundance of 11 phyla in all of the samples was <0.5%, which included *Armatimonadetes*, *Chloroflexi*, *Cyanobacteria*, *Elusimicrobia*, *Fusobacteria*, *GN02*, *LD1*, *Planctomycetes*, *SR1*, *TM7*, and *WPS2*. Among the 21 phyla, *Bacteroidetes*, *Firmicutes*, *Proteobacteria*, and *Spirochaetes* were detected as the dominant phyla among all of the samples (Table 4). The phylum *Bacteroidetes* was the most abundant in both groups (66.01% and 58.47% in the starter feed- and breast milk-fed groups, respectively), although there was no significant difference between these two groups. Within 11 phylum with an observed >0.5% relative abundance, the *Fibrobacteres (P* = 0.036), *Lentisphaerae (P* = 0.002), and *Verrucomicrobia (P* = 0.024) abundances were significantly lower in the starter feed-fed than in the breast milk-fed lambs; however, the abundance of other phyla was not significantly different between the two groups (*P* > 0.05).

Among the 17 genera observed to have >0.5% relative abundance, 5 changed significantly between the starter feed- and breast milk-fed groups (Table 5), while the others remained relatively stable. Moreover, many of the sequences (27.89% and 63.48% in the starter feed-and breast milk-fed groups, respectively) remained unclassified at the genus level. Within the *Bacteroidetes* phylum, the abundance of genus *CF231* was significantly lower in the starter feed-fed group than that in the breast milk-fed group (*P* = 0.019). Within the *Fibrobacteres* phylum, the abundance of genus *Fibrobacter* was significantly lower in the starter feed-fed group than that in the breast milk-fed group (*P* = 0.039). Within the *Firmicutes* phylum, genus *Acidaminococcus (P* = 0.004) and *Succiniclasticum (P* = 0.013) were significantly higher in the starter feed-fed group than in the breast milk-fed group. Within the *Proteobacteria* phylum, the abundance of genus *Dechloromonas* was significantly lower in the starter feed-fed group than in the breast milk-fed group (*P* = 0.050).

### OUT diversity and similarity analyses

**Figure 5:**
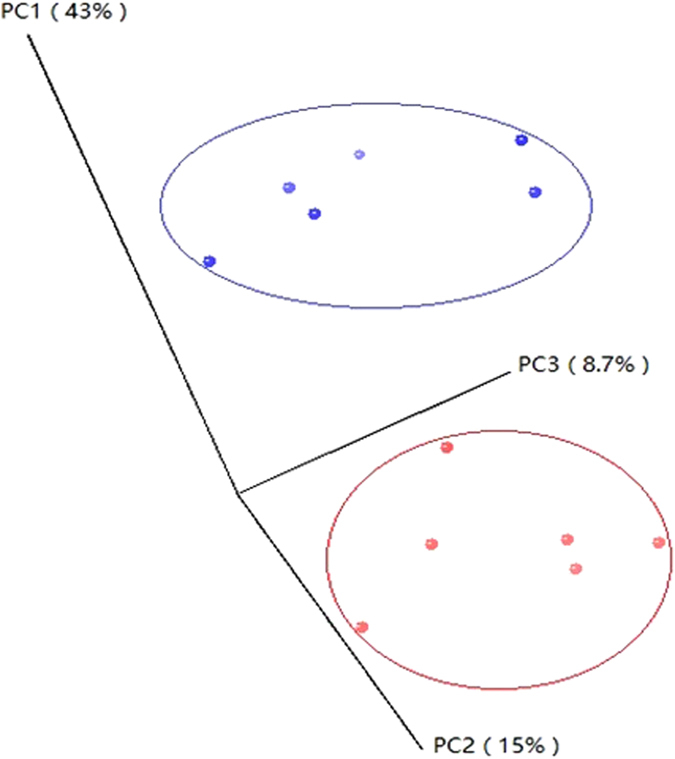
Principal coordinate analysis (PCoA) of ruminal bacterial OTUs between the starter feed- and breast milk-fed groups.

In this study, beta diversity analysis was performed. PCoA analysis using the Bray–Curtis similarity metric revealed that the samples clustered according to group (Fig. 5). PC1, PC2, and PC3 accounted for 43%, 15%, and 8.7% of variation, respectively.

### Quantification of total ruminal bacteria and abundant bacterial genera

As indicated by qPCR, the total ruminal bacterial copy numbers were higher in the starter feed-fed group than in the breast milk-fed group (*P* = 0.000). The abundance of phylum *Firmicutes (P* = 0.039) and *Bacteroidetes (P* = 0.000), and the genera *Prevotella (P* = 0.036), *Bacteroides (P* = 0.041), *and Selenomonas ruminantium-Mitsuokella multiacida (P* = 0.004) were significantly higher in the starter feed-fed group than in the breast milk-fed group (Table 6).

### Relationship between bacterial communities and functional variables

**Figure 6:**
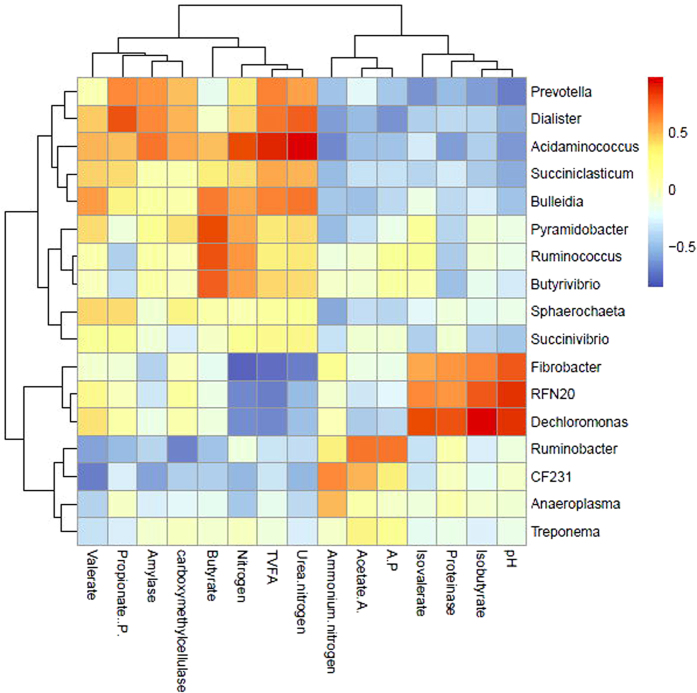
Coefficients of correlation between the relative abundances of ruminal bacterial genera and functional variables.

The abundance of bacterial communities at the genus level and functional variables were considered to be correlated with each other if the correlation coefficients were above 0.55. The relative abundance of the genera *Fibrobacter*, *RNF20*, and *Dechloromonas* were negatively correlated with the TVFA concentration, whereas *Succiniclasticum*, *Prevotella*, *Bulleidia*, *Dialister*, and *Acidaminococcus* were positively correlated with TVFA (Fig. 6). The acetate and propionate concentrations were positively correlated with the relative abundance of *Ruminobacter*, *CF231*, *Prevotella*, and *Dialister*, respectively. Butyrate concentrations were positively correlated with the relative abundances of *Ruminococcus*, *Pyramidobacter*, *Butyrivibrio*, and *Bulleidia*. The proteinase activity, isovalerate and isobutyrate concentration, and pH were all positively correlated with those of *Fibrobacter*, *RFN20*, and *Dechloromonas*. The nitrogen concentration was negatively correlated with the relative abundance of *Fibrobacter*, *RNF20*, and *Dechloromonas*, but was positively correlated with *Pyramidobacter*, *Butyrivibrio*, *Bulleidia*, and *Acidaminococcus*. Amylase activity was positively correlated with the relative abundance of *Prevotella*, *Dialister*, and *Acidaminococcus*. By contrast, the relative abundance of *Ruminobacter* was negatively correlated with carboxymethylcellulase activity, while the relative abundances of *Dialister* and *Acidaminococcus* were positively correlated with carboxymethylcellulase activity.

## Discussion

Development of the rumen is an important physiological event for young ruminants. It entails the growth and cellular differentiation of the rumen, which results in a major shift in the pattern of nutrients that are delivered to the intestines and liver, and then to the peripheral tissues of the animal^30^. Many studies have shown that the rumen development process includes anatomic development (increase in rumen mass and growth of the rumen papillae)^31^,^32^, functional achievement (fermentation capacity and enzyme activity)^33^,^34^, and microbial colonization (bacteria, fungi, archaea and protozoa)^35^,^36^. Together, these observations suggest that anatomic, functional, and microbial development in the rumen represents an integrated system, and they should be studied together to better understand the process of rumen development.

A previous study established that an early feeding strategy benefits rumen development in lambs^9^. In this present study, rumen fluid in the starter feed-fed group had greater concentrations of TVFA, and increased molar proportions of acetate, propionate, butyrate, and valerate than in the breast milk-fed group. This finding indicated that the consumption of starter from 7 days of life resulted in an earlier initiation of rumen fermentation^31^. Increased amounts of TVFA, acetate, propionate, butyrate, and valerate in the rumen of starter feed-fed lambs likely accounted for the greater WRR, VRR, and PH documented in this present study. The ruminal fluid pH value represents an important index of rumen health. Rumen pH values below 5.0 to 5.5 are considered to be abnormal and suggestive of SARA (subacute ruminal acidosis), whereas rumen pH values of 5.6 to 5.8 are considered to be marginal^37^,^38^. The ruminal fluid pH value in the starter feed-fed group was 5.3, which might be at risk for SARA. However, Li *et al*. demonstrated that SARA is not solely rumen pH-dependent, and it should be combined with clinical symptoms for diagnosis^39^. Because the starter feed-fed lambs in this study were without clinical symptoms of SARA and the development of rumen morphology in the starter feed-fed lambs was better than that in breast milk-fed lambs, we propose that the starter feed-fed lambs were not in SARA. The reason for the lower pH value in the starter feed-fed group may be the sampling time. Moreover, greater starter consumption can lead to higher amylase and carboxymethylcellulase activity potential^40^. Therefore, it is not surprising that we found that the amylase and carboxymethylcellulase activity potentials were greater in the starter feed-fed group. This finding implied that optimal fibre-degrading capacity occurred when the starter was administered^33^. In summary, the physical form of forage could accelerate rumen development in lambs.

Our present study along with previous findings have established that solid feed consumption increases total VFA concentrations in the rumen of starter feed- compared with breast milk-fed lambs. Some studies have reported that VFAs in the rumen, especially butyrate, enhance the growth of rumen papillae^3^,^6^,^7^; however, the underlying molecular mechanisms involved remain poorly characterized. In this present study, we used deep RNA sequencing to analyse the rumen transcriptomes of starter feed- and breast milk-fed lambs. We identified 225 DEGs in rumen tissues between starter feed- and breast milk-fed lambs. To investigate the biological functions of the DEGs, we performed GO annotation and KEGG pathway analysis. This study clearly revealed that most DGEs enriched many metabolism pathways, among which the most enriched categories were lipid metabolism (*P* = 2.42E-09, 331 genes, including 19 DEGs) and carbohydrate metabolism (*P* = 1.27E-06, 337 genes, including 16 DEGs). Lipid and carbohydrate metabolism may be two key pathways responsible for starter feed-dependent acceleration of rumen development in lambs.

We found that genes associated with the carbohydrate and lipid metabolism pathways were all up-regulated in starter feed-fed lambs, including *HMGCL*, *HMGCS2*, *PCK2*, and *MCEE* suggesting that rumen carbohydrate and lipid metabolism were more active in starter feed-fed lambs than in breast milk-fed lambs. *HMGCL* and *HMGCS2* were enriched in the butanoate metabolism pathway. The mature ruminal epithelium captures most of its energy from the oxidation of VFA. More than 90% of butyrate produced during microbial fermentation is oxidized and used for ketogenesis^41^. *HMGCS2* plays a central role in coordinating ruminal ketogenic flux, similar to its role in the liver^41^. *HMGCL* contributes to ketogenesis by converting HMG-CoA to acetoacetate. Ketogenesis is a hallmark of the metabolic development of ruminal epithelium tissue^42^. Based on rumen morphology and fermentation parameters, these up-regulated genes that are related to ketogenesis may be regulated by increased VFA, especially butyrate, in the rumen of starter feed-fed lambs.

The rumen readily metabolizes pyruvate, which contributes carbon for the production of oxaloacetate, lactate, or ketone bodies^43^. Zhang *et al*. reported the expression of *PCK2* mRNA transcripts was induced by propionate in a concentration-dependent manner, suggesting that propionate can directly regulate its own metabolism in young ruminant hepatocytes via the upregulation of *PCK2* mRNA transcript expression^44^. Additionally, MCEE (methylmalonyl-CoA epimerase enzyme) is found throughout nature and functions as a catabolic enzyme in higher animals^45^. Additionally, the micro-organisms that colonize animals may use this enzyme for various other functions, such as propionate fermentation and glyoxylate regeneration^46^. In this present study, expression of *PCK2* and *MCEE* mRNA transcripts and the proportion of propionate in starter feed-fed lambs were both higher than in breast milk-fed lambs. Thus, starter feed consumption can induce more propionate metabolism through the upregulation of *PCK2* and *MCEE* mRNA transcripts.

Associations between gastrointestinal microbial communities and their hosts have been recently shown to play an important role in host health and physiological function. The ruminal bacteria can digest complex and simple carbohydrates in the rumen and produce nutrients, such as volatile fatty acids (VFA), microbial proteins, and vitamins, for the host. Diet is a major factor that influences the structure and function of microbial communities in the rumen^14^. In this present study, we elucidated changes in the rumen microbial taxonomical composition in starter feed- and breast milk-fed lambs at the phylum and genus levels. Using the Roche-454 Titanium platform, we obtained an average of 15,780 reads for each sample with good coverage (>97.3%). Furthermore, our findings indicate that each group of lambs has a distinct ruminal microbiota, as is reflected by the clustering of samples between breast milk- and starter feed-fed lambs using PCoA. Moreover, most alpha-diversity indices (except the Simpson index) were higher in the breast milk-fed group than in the starter feed-fed group, suggesting that the ruminal microbiota in the breast milk-fed group is more diverse than in the starter feed-fed group.

It is commonly thought that a more diverse rumen microflora promotes greater stability in the rumen environment. However, the establishment of ruminal bacterial communities in the first days after birth might involve a more complex process. Li *et al*. reported that the rumen microbiota of younger calves (14 days) exhibited a heterogeneous microbial composition and contained more numerous, yet transient bacterial species and genera than older calves (42 days). Moreover, a significantly higher percentage of input 16S sequence reads from microbiota from younger calves could be assigned to any genus, indicating that more unknown bacteria may exist in the microbiome of younger calves^47^. This possibility is similar to that of the breast milk-fed lambs in this study, indicating that early starter feeding promotes the establishment of predominant microflora and the depletion of transient bacterial species and genera. In this present study, only 3 of the bacterial genus that disappeared in the starter feed-fed group but not in the breast milk-fed group have a fermentation function; most of the ‘disappeared’ bacterial genus are aerobic bacteria or bacteria without fermentation function. Jami *et al*. reported that the most significant change occurred between the first and third days of life in the rumen was the reduction in taxa associated with aerobic or facultative anaerobic function and an increase in those taxa associated with obligatory anaerobic function. Furthermore, almost all genera that showed a sharp reduction on day 3 were either aerobic or facultative anaerobic, indicating the emergence of a new anaerobic environmental niche as early as 3 days after birth^48^. However, in this present study, anaerobic environment of the rumen is not yet fully established in breast milk-fed lambs by 42 days of age, as many aerobic or facultative anaerobic bacterial genus still remain. These observations indicate that early starter feeding promotes the establishment of the ruminal anaerobic environment and stability of the rumen microflora.

In both groups, *Bacteroidetes*, *Firmicutes*, *and Proteobacteria* were the dominant phyla in ruminal microbiota, in accord with previous studies^49^,^50^,^51^. As previously reported, *Verrucomicrobia* could be found at a higher proportion in calves exclusively fed milk and were represented exclusively by the genus *Akkermansia*^26^,^48^; we also found that the relative abundance of *Verrucomicrobia* was significantly higher in breast milk-fed lambs. This observation may reflect an opportunistic capacity for this phylum in young lambs. The genus *Prevotella* was prominent in starter feed-fed lambs, reaching up to 40.37% of total reads, whereas in breast milk-fed lambs the relative abundance was only 17.20%. As previously reported, *Prevotella* is the most abundant genus in the adult rumen^52^ and is thought to account for a large portion of the rumen microbial genetic and metabolic diversity^53^. Jami *et al*. reported that the *Bacteroidetes* phylum is less abundant when high-calorie diets are consumed, and its composition changes to being predominantly composed of genus *Prevotella* when high-fibre diets are introduced^48^. As described above, differences in the abundance of prominent genus between starter feed- and breast milk-fed groups of lambs could be a consequence of differences in fermentable substrates in the rumen.

Based on the data on rumen function variables, we explored the relationship between ruminal microbiota and rumen functions. The abundances of the genera *Prevotella*, *Bulleidia*, *Dialister*, and *Acidaminococcus* were each found to be positively correlated with TVFA and urea nitrogen concentrations, while the abundances of the genera *Fibrobacter*, *RFN20*, and *Dechloromonas* were each negatively correlated with TVFA and nitrogen concentrations, suggesting that they might be involved in nitrogen and volatile fatty acid metabolism. Similarly, the genera *Dialister* and *Acidaminococcus* might also participate in fibrolytic enzyme secretion and starch degradation.

Although ruminal microbiota in breast milk-fed lambs was more diverse than in starter feed-fed lambs, the absolute abundance of total bacteria in starter feed-fed lambs was markedly higher than in breast milk-fed lambs, indicating that the amount of fermentable substrate is critical for bacterial proliferation. Early starter feeding provides ample organic matter for micro-organism fermentation, thereby promoting the reproduction of bacteria and establishment of predominant microflora, while increasing the abundance of fermented products, such as VFA, NH_3_, and other small molecules. As revealed by the results of RNA-seq in this present study, fermented products in the rumen enhance the growth of rumen papillae by affecting many metabolic pathways, including lipid and carbohydrate metabolism.

Thus, based on the overall mRNA expression profiles and abundance of bacteria, we conclude that improvements in rumen morphology and function in starter feed-fed lambs are a consequence of stimulation of ruminal ketogenesis and butanoate metabolism via the *HMGCL* and *HMGCS2* genes combined with changes in the fermentation type because of the ruminal microbiota. Confirmation of a causative relationship between gene expression and rumen development stimulated by starter feeding in sheep may require further studies that use knock-in or knock-out animals to up- or down-regulate the expression of related genes.

## Conclusion

Overall, we identified 225 DEGs in the rumen between starter feed- and breast milk-fed lambs. Functionally, these genes were related to metabolism, particularly of lipids and carbohydrates. The genes *HMGCL* and *HMGCS2* were enriched in the butanoate metabolism pathway and play critical roles in ketogenesis. Additionally, the rumen microbiota of pre-ruminant lambs was responsive to dietary modifications, as well as structural and physiological changes in the host. The ruminal bacterial communities were more diverse in the breast milk-fed group than in the starter feed-fed group, as each group had a distinct microbiota. After starter feed consumption, many aerobic bacteria or non-fermenting bacteria disappeared. We propose that early administration of starter feed is beneficial to the development of rumen morphology and function in lambs, and the underlying mechanism may involve the stimulation of ruminal ketogenesis and butanoate metabolism via the *HMGCL* and *HMGCS2* genes combined with changes in the fermentation type mediated by the ruminal microbiota. This study enhances our understanding of the molecular mechanism that regulates rumen development in sheep.

## Materials and Methods

### Ethics Statement

All experiments in this study were carried out in accordance with the approved guidelines from the Regulation of the Standing Committee of Gansu People’s Congress. All experimental protocols and the collection of samples were approved by the Ethics Committee of Gansu Agricultural University.

### Animals and sample collection

A total of 12 purebred male Hu lambs were used in this study that were obtained from a commercial sheep farm (Jinchang Zhongtian Sheep Industry Co., Gansu, China). Lambs weighed 3.51 ± 0.57 kg at birth. The beginning date of experimentation was adjusted for each lamb to account for different birth dates. To ensure that environmental conditions were similar throughout the experiment, all lambs were housed in a well-ventilated room with controlled temperature and humidity. The 12 lambs were divided into 2 groups (breast milk- and starter feed-fed groups) under the homogeneity principle (6 lambs per group). Starter feed-fed lambs received the starter diet (Table S7) from day 7 of life. Ewes were fed the diet three times per day at 06:30–08:30, 12:30–14:30, and 18:00–20:00. When feeding ewes, the lambs and ewes were separated and the lambs were fed a starter diet, but they did not touch the ewes’ feed. After ewe feeding, the feeders were removed. Lambs were released from the feeding fence, and were fed the starter diet *ad libitum* with free access to water. Breast milk-fed lambs only suckled milk without receiving the starter diet. Body weights were measured before the morning feeding. All lambs were slaughtered at 42 days according to a standard procedure that was approved by the Biological Studies Animal Care and Use Committee, Gansu Province, P.R. of China.

For RNA sequencing, the rumen tissues of three starter feed-fed lambs and three breast milk-fed lambs were selected by referring to the index of rumen morphology, rumen fermentation parameters, and enzymatic activity of rumen micro-organisms. For rumen microbial 16S rRNA sequencing, 6 starter feed-fed lambs and 6 breast milk-fed lambs were selected. Immediately after slaughter, a section of rumen tissue from the ventral blind sac (2 × 2 cm in size) was quickly excised. Tissue samples were snap-frozen in liquid nitrogen and stored at –80 °C for subsequent total RNA analysis. Rumen fluid was collected and strained through four layers of cheesecloth, transferred into plastic bottles, and the stored at −80 °C for subsequent DNA analysis.

### Rumen morphology

After slaughter, the rumen was separated and ligatured using cotton thread. The displacement method was used to measure the rumen volume. The rumen weight was measured after cleaning and eliminating the rumen contents. Rumen tissue specimens (~1 × 1 cm) were obtained from the cranial dorsal sac and fixed in 40% formaldehyde. After fixation, tissue specimens were trimmed and processed according to standard histological procedures, then were stained with haematoxylin and eosin. For each tissue specimen, a total of three sections with 5+ papillae length (PL), papillae width (PW), and muscular layer thickness (ML) were examined. PL and PW were measured in well-oriented papillae for each rumen sac, while the ML was measured at random locations in each rumen sac. PL was defined as the distance from the tip to the base of the papillae, while the PW was defined as the average width of the papillae base, middle, and tip. Morphometric analyses were performed at a magnification of 4 × 10 times (Olympus BX-51; Olympus Corporation, Tokyo, Japan) using Image Pro plus 6.0 (Media Cybernetics, Silver Spring, MD, USA).

### Volatile fatty acid assay

The ruminal fluid pH was measured immediately after collection using a digital pH meter (PB21, Sartorius, Goettingen, Germany). Individual and total VFA in aliquots of strained ruminal fluid were quantified by gas chromatography^54^. Samples were injected using an auto-sampler (AI 3000, Thermo Scientific, Waltham, MA, USA) into an AE-FFAP capillary column (30 m × 0.25 mm × 0.33 μm, ATEO, LanZhou, China) on a Varian GC (TRACE 1300, Thermo Scientific, MA, USA). Samples were run at a split-ratio of 20:1 with a column temperature of 45 °C to 150 °C with an increase of 10 °C/min followed by a 5-min hold. The injector and detector temperatures were 200 °C and 250 °C, respectively. Peak integration was performed using Chromeleon^®^ Software. All ruminal fluid samples were assayed in duplicate.

### Enzymatic activity assay

Samples for enzyme activity potential measurements were prepared as suggested by Rey *et al*.^34^. Enzyme activity potentials of carboxymethylcellulase and amylase in rumen contents were determined by measuring the release of reducing sugars from substrates (carboxymethylcellulose and starch, respectively)^34^; reaction times were 30, 15, and 15 min, respectively. One enzyme activity unit (U) was defined as the amount of enzyme required to release 1 μmol reducing sugars (xylose or glucose equivalents)/min per g of wet rumen content^55^. Protease activity was assayed using azocasein as a substrate, according to the method of Eun and Beauchemin^56^. In this assay, the hydrolysis of azocasein released an azo group, which induced a colour change that could be measured by spectrophotometry at 420 nm.

### RNA preparation and sequencing

Total RNA was extracted using TransZol reagent (TransGen Biotech, Beijing, China) according to the manufacturer’s instructions. Paired-end libraries were prepared for each RNA-seq sample using a “Truseq^®^ Stranded Total RNA Sample Preparation Kit (Illumina^®^)”; all of these procedures were performed according to the manufacturer’s instructions. After quality control, sequencing of all the libraries was performed using an Illumina HiSeq 2500 instrument. RNA library construction and sequencing was performed by Shanghai Personal Biotechnology Co., Ltd.

### Analyses of RNA-Seq data

The RNA-Seq results were transferred from an Illumina fastq format to a standard Sanger fastq format with fq_all2std.pl; data were processed using the Tophat–Cufflinks pipeline^57^. The *Ovis aries* reference genome and gtf annotation file were downloaded from Ensembl (*O. aries* 3.1) and the build index was implemented with bowtie version 2.1.0. TOPHAT (version 2.0.9) was used for transcriptome assembly, and the Cuffdiff script from Cufflinks was used for gene expression analysis with the option-classic-fpkm. Expression levels of each gene were represented by the FPKM value, which indicates the fragments per kilobase of exon per million fragments mapped, and was calculated by the following formula^58^:


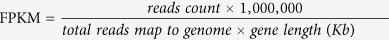


Finally, genes were classified as differentially expressed if they exhibited two-fold or greater changes between the starter feed- and breast milk-fed groups, and if they showed statistical significance at p ≤ 0.05 based on the Audic–Claverie method^59^,^60^,^61^ with a false discovery rate (FDR) < 0.05^62^.

### Cluster analysis

Hierarchical clustering analysis was carried out for all annotated transcripts from the breast milk- and starter feed-fed groups. The RPKM counts for each transcript were clustered using the Cluster 3.0 software package, and JAVA Treeview was used to view the cluster images. Results were visualized using JAVA Treeview^63^.

### Gene ontology and KEGG enrichment analysis of differentially expressed genes

The functional categories of DE genes were established with the GO (Gene Ontology) and KEGG (Kyoto Encyclopedia of Genes and Genomes). GO and KEGG enrichment analyses were performed via http://www.geneontology.org and http://www.genome.jp/kegg/. Analyses were conducted as described by Ashburner *et al*.^64^ and Kanehisa *et al*.^65^.

### Q-RT-PCR validation of DE genes

Relative expression levels of DE genes in the rumen were quantified by real-time PCR. *GAPDH* was selected as an internal control for qRT-PCR validation because of the stable expression of its mRNA transcript in rumen tissues. The primer sequences and PCR conditions for analysed genes are listed in Supplementary Table S8. The 2^−ΔΔCt^ method was used for relative gene expression level analysis^66^. For each gene, the average ΔCt value of the breast feed-fed group was used as reference to calculate the −ΔΔCt value, and Student’s *t*-test was used to analyze expression differences between two groups. Correlations were calculated by Pearson’s correlation (SPSS 16.0, SPSS Inc., Chicago, IL, USA), using data from RNA-seq and qRT-PCR.

### DNA extraction, PCR amplification, and sequencing

Total genomic DNA was isolated from rumen contents using the Omega E.Z.N.A^TM^ Stoll DNA Kit according to the manufacturer’s instructions (Omega Bio-Tek, USA). Concentrations of extracted DNA were determined using a Nano-Drop 2000 spectrophotometer (Thermo Scientific, Wilmington, DE, USA). The V1-V3 hypervariable region of the bacterial 16S ribosomal RNA gene was amplified by PCR from microbial genomic DNA that had been harvested from rumen fluid samples using barcoded fusion primers (forward: 5′-AGAGTTTGATCCTGGCTCAG-3′; reverse: 5′-TTACCGCGGCTGCTGGCAC-3′)^67^. PCR thermo cycling conditions were 94 °C for 5 min; 94 °C for 30 sec, 55 °C for 60 sec and 72 °C for the extension time, repeated for 30 cycles; 72 °C 7 min. PCR products were excised from a 1.5% agarose gel and purified using a QIAquick Gel Extraction Kit (QIAGEN, cat #28706). Barcoded V1-V3 amplicons were sequenced using the Roche GS FLX + platform (454 Life Sciences-A Roche Company, Branford, CT, USA).

### Analysis of 16S rRNA sequencing data

Raw sequences were filtered through a quality control pipeline using the Quantitative Insight into Microbial Ecology (QIIME) tool kit^68^ and mother^69^ and bases with quality scores above 30 were retained for further analyses. High-quality reads were assigned to operational taxonomic units (OTUs) at a 97% identity threshold using the QIIME Uclust algorithm^68^, and taxonomical groups were assigned using the mother-based implementation of the RDP Bayesian classifier (http://rdp.cme.msu.edu/) with a 0.80 confidence threshold^70^. Chimeric DNA sequences were detected using UCHIME and removed^71^. Alpha-diversity values were obtained using various diversity indices (observed species, the Chao estimate, abundance-based coverage estimator [ACE], the Shannon and Simpson diversity indices). Principal coordinate analysis (PCoA) of microbial communities was performed using the Bray–Curtis distance^72^.

### Quantification of total bacteria and selected bacterial genera

Absolute quantitative real-time PCR (qPCR) was performed to measure copy numbers of the 16S rRNA genes of total bacteria and six selected bacterial genera (*Prevotella spp.*, *Clostridium butyricum*, *Bacteroides spp.*, *Firmicutes*, *Bacteroidetes*, and *Selenomonas ruminantium-Mitsuokella multiacida*). The primers, which were previously validated, are listed in Table S9 in the Supplemental material. All qPCR assays were performed using SYBR Premix Ex Taq (Perfect Real Time; TaKaRa, Japan) on an ABI 7900HT Fast real-time PCR system (Applied Biosystems, Foster City, CA, USA). Standard curves for total bacteria and for each bacterial genus were prepared using plasmid DNA that contained each unique 16S rRNA insert. The copy number of the 16S rRNA gene per gram of fresh tissue was calculated as described by Zhou *et al*.^73^. The relative abundances of bacterial genera were calculated by dividing the copy number of the 16S rRNA gene for each genus by the copy number of the 16S rRNA genes for total bacteria.

### Correlations between ruminal bacterial communities and anatomic and functions variables

Spearman’s rank correlations between ruminal bacterial communities (Roch 454 relative abundance) and anatomic and functions variables were analysed using the PROC CORR procedure of SAS. Only those bacterial groups that represented >2% of the total community in at least one sample and that were detected in >50% of the rumen tissue samples were included in the analysis^29^.

### Statistical analysis

Differences in growth performance, rumen morphology, rumen fermentation parameters, and the enzymatic activity of rumen micro-organisms between the beast milk- and starter feed-fed groups were analysed using SPSS Version 16.0 for Windows (SPSS, Chicago, IL, USA). A one-way analysis of variance and the least significant difference (LSD) *t*-test were used for multiple comparisons. A P value of <0.05 was considered to indicate a statistically significant difference. Data are presented as means ± standard deviation.

### Nucleotide sequence accession numbers

All RNA sequence reads have been submitted to the NCBI BioProject under accession code PRJNA317746 and PRJNA317540. All 16S rRNA sequence reads have been submitted to the NCBI BioProject under accession code PRJNA317793.

## Additional Information

**How to cite this article**: Wang, W. *et al*. Effects of early feeding on the host rumen transcriptome and bacterial diversity in lambs. *Sci. Rep.* **6**, 32479; doi: 10.1038/srep32479 (2016).

## Supplementary Material

Supplementary Information

Supplementary Information

Supplementary Information

Supplementary Information

Supplementary Information

Supplementary Information

Supplementary Information

Supplementary Information

Supplementary Information

Supplementary Information

## Figures and Tables

**Table 1:** Animal performance of Hu sheep used for RNA and metagenomics sequencing.

Item			Breast milk-fed	Starter feed-fed	*P*-value	
Growth performance	BW (kg)		8.49 ± 1.94	12.05 ± 2.72	0.026	
	ADG (g/d)		0.13 ± 0.01	0.18 ± 0.01	0.010	
Rumen morphology	WRR (g)		98.32 ± 27.59	196.72 ± 35.62	**<0.001**	
	VRR (mL)		894.6 ± 320.86	1500.7 ± 291.92	**0.007**	
	PH (μm)		659.40 ± 19.97	1003.33 ± 24.95	**<0.001**	
	PW (μm)		624.74 ± 40.02	474.90 ± 18.79	0.053	
	MT (μm)		807.45 ± 35.26	849.62 ± 39.59	0.429	
Rumen fermentation parameters	pH		5.91 ± 0.10	5.30 ± 0.04	0.130	
	Total VFA (mmol/L)		33.05 ± 5.52	113.24 ± 7.99	**<0.001**	
	VFA proportion (mol/100 mol)	Acetate (A)	58.45 ± 9.24	50.67 ± 5.64	**0.015**	
		Propionate (P)	26.98 ± 5.13	32.05 ± 6.63	**0.025**	
		Butyrate	8.65 ± 1.50	11.78 ± 2.66	**<0.001**	
		Isobutyrate	0.97 ± 0.81	0.31 ± 0.23	**0.011**	
		Valerate	2.63 ± 1.21	3.84 ± 0.96	**0.007**	
		Isovalerate	2.32 ± 2.07	1.34 ± 1.22	0.152	
		A:P	2.32 ± 0.88	1.67 ± 0.49	**0.028**	
	ruminal metabolic parameters (mg/100 mL)	Nitrogen	929.44 ± 290.70	4267.08 ± 558.99	**<0.001**	
		Ammonium nitrogen	13.48 ± 3.55	22.66 ± 1.70	**<0.001**	
		Urea nitrogen	2.68 ± 0.04	8.97 ± 1.44	**<0.001**	
		Protein nitrogen	913.29 ± 236.48	4235.44 ± 423.10	**<0.001**	
Enzymes activity of rumen microorganisms	Proteinase (U/mg)		0.017 ± 0.001	0.007 ± 0.000	**<0.001**	
	Amylase (U/mg)		0.294 ± 0.021	0.585 ± 0.034	**<0.001**	
	carboxymethylcellulase (U/mg)		0.019 ± 0.002	0.034 ± 0.001	**<0.001**	

BW – body weight. ADG –average daily gain. WRR –weight of the reticulo-rumen. VRR –volume of the reticulo-rumen. PH –papilla height of the rumen. PW –papilla width of the rumen. MT –muscular thickness of the rumen. *P*-values were calculated using Student’s *t*-test.

**Table 2:** List of 20 rumen DEGs between Hu sheep that received starter feed or breast milk.

Gene	Starter feed-fed	Breast milk-fed	FC (S/B)	*P*-value	Full name
*GPNMB*	35.30	1998.39	−56.61	4.76E-02	Glycoprotein (transmenbrane) nmb
*KRT4*	92.20	4519.14	−49.01	3.64E-04	Keratin 4, type II
*Unknown1*	4.04	110.80	−27.42	1.06E-02	—
*TRDC*	1.23	31.40	−25.57	4.34E-02	T cell receptor delta constant
*Unknown2*	1.55	36.35	−23.43	4.14E-02	—
*SRGN*	2.85	58.12	−20.41	1.91E-02	Serglycin
*RGS4*	7.71	148.34	−19.23	1.41E-02	Regulator of G-protein signaling 4
*FGR*	2.20	40.02	−18.19	4.80E-02	FGR proto-oncogene, Src family tyrosine kinase
*NXPE2*	2.80	50.11	−17.88	9.10E-03	Neurexophilin and PC-esterase domain family, member 2
*Unknown3*	332.15	4734.76	−14.25	2.02E-06	—
*COX8C*	1.86	229.54	123.39	3.03E-07	Cytochrome c oxidase subunit VIIIC
*Unknown4*	0.23	32.03	137.98	1.20E-02	—
*MYL10*	0.31	53.46	172.41	1.58E-02	Myosin, light chain 10, regulatory
*Unknown5*	182.28	31458.78	172.59	6.24E-03	—
*Unknown6*	242.21	56941.99	235.09	6.97E-03	—
*Unknown7*	5.05	1532.46	303.38	1.25E-02	Ribosomal protein
*Unknown8*	1.20	372.79	311.68	2.13E-02	—
*Unknown9*	0.65	239.04	366.04	3.50E-02	—
*Unknown10*	1.23	626.32	509.61	5.94E-03	—
*Unknown11*	0.31	295.88	954.32	4.82E-02	—

**Table 3:** Alpha diversity measures of bacterial communities in the ruminal contents between the starter feed- and breast milk-fed groups.

Alpha diversity index	Breast milk-fed	Starter feed-fed	*P*-value
OTU	1601.7 ± 108.5	798.0 ± 65.6	<0.001
Chao	1940.8 ± 110.8	972.7 ± 65.1	<0.001
ACE	2045.8 ± 117.1	1019.3 ± 67.6	<0.001
Simpson	0.024 ± 0.004	0.104 ± 0.031	0.028
Shannon	5.560 ± 0.172	3.878 ± 0.283	<0.001
Coverage	0.961 ± 0.002	0.986 ± 0.002	<0.001

**Table 4:** Phylum-level taxonomic composition of bacterial communities in the ruminal contents between the starter feed- and breast milk-fed groups.

Phylum	% of sequences in starter feed-fed group	% of sequences in breast milk-fed group	*P*-value
*Actinobacteria*	0.132 ± 0.072	0.335 ± 0.226	0.413
*Bacteroidetes*	66.007 ± 4.187	58.471 ± 1.385	0.118
*Fibrobacteres*	0.084 ± 0.050	0.769 ± 0.279	**0.036**
*Firmicutes*	19.367 ± 3.551	14.99 ± 1.484	0.283
*Lentisphaerae*	0.119 ± 0.088	3.265 ± 0.752	**0.002**
*Proteobacteria*	2.593 ± 1.231	6.016 ± 1.441	0.101
*Spirochaetes*	4.690 ± 1.202	9.583 ± 4.780	0.344
*Synergistetes*	1.200 ± 0.450	1.070 ± 0.529	0.862
*Tenericutes*	0.677 ± 0.004	1.186 ± 0.335	0.363
*Verrucomicrobia*	0.149 ± 0.146	7.481 ± 2.762	**0.024**
Unclassified	0	0.003 ± 0.002	0.154
Others (<0.5%)	0.088 ± 0.067	1.715 ± 0.290	<0.001

**Table 5:** Genus-level taxonomic composition of the bacterial communities in the ruminal contents between the starter feed- and breast milk-fed groups.

Phylum	Genus	% of sequences in starter feed-fed group	% of sequences in breast milk-fed group	*P*-value
*Bacteroidetes*	*Prevotella*	40.370 ± 9.576	17.196 ± 5.925	0.067
	*CF231*	0.064 ± 0.000	1.613 ± 0.553	**0.019**
*Fibrobacteres*	*Fibrobacter*	0.084 ± 0.050	0.750 ± 0.277	**0.039**
*Firmicutes*	*Butyrivibrio*	0.821 ± 0.241	0.328 ± 0.075	0.079
	*Ruminococcus*	3.240 ± 2.658	0.441 ± 0.123	0.318
	*Acidaminococcus*	1.580 ± 0.426	0.001 ± 0.001	**0.004**
	*Dialister*	3.721 ± 1.777	0.001 ± 0.001	0.063
	*Succiniclasticum*	5.043 ± 1.399	0.765 ± 0.223	**0.013**
	*Bulleidia*	2.512 ± 1.875	0.011 ± 0.006	0.210
	*RFN20*	0.006 ± 0.006	0.923 ± 0.473	0.081
*Proteobacteria*	*Dechloromonas*	0.014 ± 0.007	1.420 ± 0.632	**0.050**
	*Ruminobacter*	0.001 ± 0.001	0.864 ± 0.549	0.147
	*Succinivibrio*	1.981 ± 1.178	1.014 ± 0.988	0.544
*Spirochaetes*	*Sphaerochaeta*	6.617 ± 4.016	1.364 ± 0.537	0.224
	*Treponema*	2.964 ± 2.951	3.187 ± 1.423	0.947
*Synergistetes*	*Pyramidobacter*	1.048 ± 0.490	0.213 ± 0.060	0.122
*Tenericutes*	*Anaeroplasma*	0	0.567 ± 0.325	0.112
Unclassified (Genus)		27.919 ± 8.595	53.156 ± 6.044	**0.037**
Others (<0.5%)		0.019 ± 0.004	0.157 ± 0.027	**0.000**

**Table 6:** qPCR results for total bacteria, two phyla, and four genera of ruminal bacterial communities in the starter feed- and breast milk-fed groups.

Taxon	Abundance of bacteria^a^		*P-*value
	Starter feed-fed group	Breast milk-fed group	
*Bacteria*	30.76 ± 2.77	5.23 ± 1.25	**<0.001**
*Bacteroidetes*	16.38 ± 1.13	3.12 ± 0.77	**<0.001**
*Firmicutes*	3.90 ± 1.24	0.87 ± 0.30	**0.039**
*Prevotella*	11.53 ± 4.46	0.66 ± 0.43	**0.036**
*Clostridium Butyricum*	0.22 ± 0.20	0.0015 ± 0.00075	0.285
*Bacteroides spp.*	0.02 ± 0.004	0.009 ± 0.003	**0.041**
*Selenomonas ruminantium-Mitsuokella multiacida*	0.195 ± 0.052	0.0017 ± 0.00083	**0.004**

^a^The abundance of total bacteria is expressed as 10^10^ copy numbers per gram of rumen content.
