# Supplementary material for: Effects of early feeding on the host rumen transcriptome and bacterial diversity in lambs
Source: Sci Rep. 2016 Aug 31;6:32479. doi: 10.1038/srep32479 (PMC5006043; doi:10.1038/srep32479)

**Supplementary Information**

**Title**

Effects of early feeding on the host rumen transcriptome and bacterial diversity in lambs

**Authors**

Weimin Wang, Chong Li, Fadi Li*, Xiaojuan Wang, Xiaoxue Zhang, Ting Liu, Fang Nian, Xiangpeng Yue, Fei Li, Xiangyu Pan, Yongfu La, Futao Mo, Fangbin Wang, Baosheng Li

* the corresponding author

**Supplementary Figures**

**Figure S1 Characterization of RNA-seq mapped reads in rumen from starter feed- and breast milk-fed lambs.**


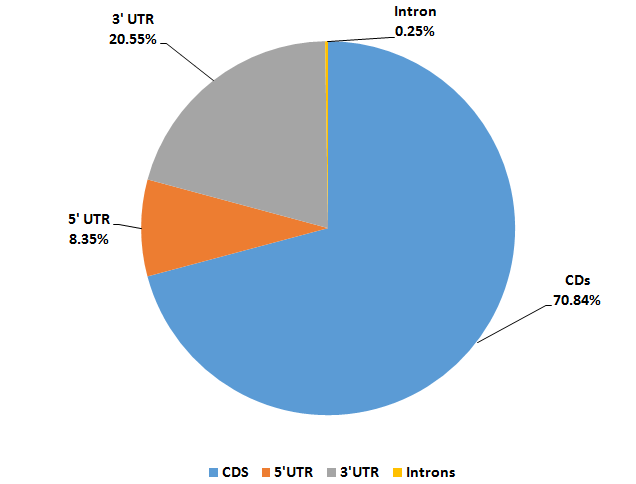


**Figure S2 Rarefaction curve (number of OTUs) of the rumen content from starter feed- and breast milk-fed lambs.**


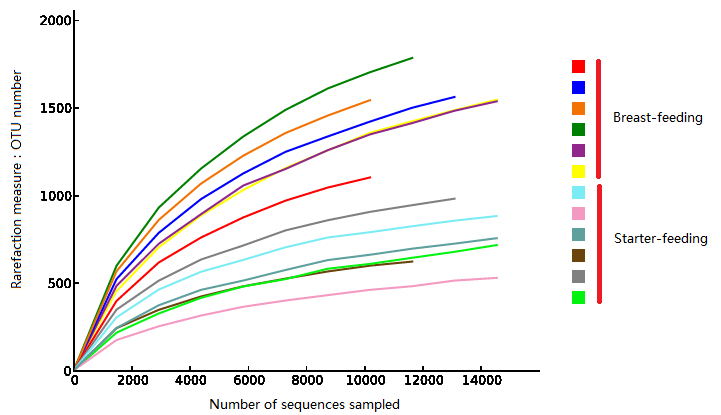

Supplement: Supplementary Information [file srep32479-s1.doc]
